# *Populus* root exudates are associated with rhizosphere microbial communities and symbiotic patterns

**DOI:** 10.3389/fmicb.2022.1042944

**Published:** 2022-12-22

**Authors:** Mengjie Li, Zhen Song, Zhanbiao Li, Rongye Qiao, Pingdong Zhang, Changjun Ding, Jianbo Xie, Yinglong Chen, Hui Guo

**Affiliations:** ^1^College of Biological Sciences and Technology, Beijing Forestry University, Beijing, China; ^2^Institute of Environment and Sustainable Development in Agriculture, Chinese Academy of Agricultural Sciences, Beijing, China; ^3^State Key Laboratory of Tree Genetics and Breeding, Key Laboratory of Tree Breeding and Cultivation of State Forestry Administration, Research Institute of Forestry, Chinese Academy of Forestry, Beijing, China; ^4^UWA School of Agriculture and Environment, UWA Institute of Agriculture, Perth, WA, Australia; ^5^National Engineering Research Center of Tree Breeding and Ecological Restoration, Beijing, China

**Keywords:** dominant taxa, keystone taxa, differential root exudates, phenolic compounds, interactive effect

## Abstract

**Introduction:**

Microbial communities in the plant rhizosphere are critical for nutrient cycling and ecosystem stability. However, how root exudates and soil physicochemical characteristics affect microbial community composition in *Populus* rhizosphere is not well understood.

**Methods:**

This study measured soil physiochemistry properties and root exudates in a representative forest consists of four *Populus* species. The composition of rhizosphere bacterial and fungal communities was determined by metabolomics and high-throughput sequencing.

**Results:**

Luvangetin, salicylic acid, gentisic acid, oleuropein, strigol, chrysin, and linoleic acid were the differential root exudates extracted in the rhizosphere of four *Populus* species, which explained 48.40, 82.80, 48.73, and 59.64% of the variance for the dominant and key bacterial or fungal communities, respectively. Data showed that differential root exudates were the main drivers of the changes in the rhizosphere microbial communities. *Nitrosospira*, *Microvirga*, *Trichoderma*, *Cortinarius*, and *Beauveria* were the keystone taxa in the rhizosphere microbial communities, and are thus important for maintaining a stable *Populus* microbial rhizosphere. The differential root exudates had strong impact on key bacteria than dominant bacteria, key fungi, and dominant fungi. Moreover, strigol had positively effects with bacteria, whereas phenolic compounds and chrysin were negatively correlated with rhizosphere microorganisms. The assembly process of the community structure (keystone taxa and bacterial dominant taxa) was mostly determined by stochastic processes.

**Discussion:**

This study showed the association of rhizosphere microorganisms (dominant and keystone taxa) with differential root exudates in the rhizosphere of *Populus* plants, and revealed the assembly process of the dominant and keystone taxa. It provides a theoretical basis for the identification and utilization of beneficial microorganisms in *Populus* rhizosphere.

## Introduction

Soil microorganisms play an important role in plant growth. Beneficial microbial population can effectively promote the nutrient utilization, growth, and development of plants, and improve plant stress resistance (Song et al., 2020; Gupta et al., 2021). Early studies revealed more abundant microbial density, species richness, and metabolic activity in the rhizosphere than in the bulk soil, which has been regarded as “rhizosphere effect” (Ma et al., 2021). Various rhizosphere microorganisms, such as *Pseudomonas*, *Streptomyces*, and *Fusarium*, are common in the plant habitat (Wu N. et al., 2021). Microbe-microbe interactions sustain ecological balance. Network comprises the various interactions, including complex positive (e.g., commensalism and mutualism) and negative (e.g., predation and competition) interactions (Jiang Y. et al., 2017). For example, reconstructing a consortium of *Chitinophaga* and *Flavobacterium* consistently suppresses root disease caused by *Rhizoctonia solani* (Carrión et al., 2019). *Pseudomonas* interacts with *Lysobacter* and *Bacillus* to colonize the plant rhizosphere, where they supply nutrients for plant growth (Jiang Y. et al., 2017). In-depth research of co-occurrence networks is essential to understand the underlying interactive effect of the microbial communities, and to identify possible keystone populations in the communities.

Microbial groups (dominant and key microorganisms) play essential roles in promoting the utilization of nutrient elements by plant. Rhizosphere microorganisms are affected by soil nutrients and pH, electrical conductivity (EC), and soil moisture (Jiang J. et al., 2017; Bahram et al., 2018; Bai et al., 2020; Zhou et al., 2020). The composition, diversity, and dominance of bacteria vary among soils with different pH and soil organic carbon content (Sui et al., 2021). Basal microbial respiration depends strongly on soil moisture because of the water’s crucial role in substrate diffusion (Kundel et al., 2020); in turn, this directly affects the cycling of nutrients to microbes in forest ecosystems, including carbon cycling and nitrogen transformation (Wagg et al., 2014).

In addition to soil physicochemical properties, root exudates also affect the composition of the rhizosphere microbial communities. Root exudates account for 5–21% of the total photosynthetically fixed carbon, including organic acids, fatty acids, phenolics, amino acids, polysaccharides, and other secondary metabolites. The microbes make use of root exudates as nitrogen and carbon sources, and as signal stimuli (Ma et al., 2021). Phenolics are the most abundant plant metabolites and have been used as a slow carbon pool in soil dynamics models (Min et al., 2015). In *Populus*, the phenol salicylic acid acts as an inducible defense chemical that varies with tree genotype (Veach et al., 2019). Several microbial groups interact with salicylic acid, including Chloroflexi and the Basidiomycete family Hymenogastraceae (Veach et al., 2019). Strigol stimulates spore germination and hyphal growth of arbuscular mycorrhizal fungi (AMF). A positive correlation was detected between the capacity of a microbe to utilize compounds exuded by roots and its relative abundance therein (Rozpądek et al., 2018). During plant evolution, rhizosphere microorganisms affected root exudation. Beneficial microorganisms promote the accumulation of organic and amino acids, or produce peroxidase, which improves *Populus* plant growth under stress (Veach et al., 2020). Auxin produced by plant rhizosphere microbiota modulates flowering time and further stimulates exudation *via* a positive feedback mechanism (Lu et al., 2018). Therefore, root exudates affect the microbial community structure and microbial interactions. However, compared to the well-studied effect of nutrients on microorganisms, the role of the interaction between root metabolites and rhizosphere microorganisms in maintaining the stability of the ecosystem is not understood.

*Populus* is a genus of the Northern Hemisphere willow family (Salicaceae), and is the model organism for the study of woody perennials that may serve as an ideal model for understanding plant-microbe interactions (Feng et al., 2013). The growth and development of *Populus* are partially dependent on the functional microbial communities in the rhizosphere, which is the critical root-soil interface for plant growth (Song et al., 2020). Research on *Populus* has focused on plant aboveground traits, such as photosynthetic characteristics, carbon and nitrogen storage, and circulation, or focused on single factor on the belowground traits (Hogan et al., 2021; Wu X. et al., 2021; Wang et al., 2022). While research on the belowground traits, including dominant combined keystone taxa in the rhizosphere of *Populus* and the regulatory effects of differential root exudates on dominant and keystone taxa is lacking. Thus, this study used multi-omics technique (genomic-metabolomics) to explore microbial communities (dominant and key microorganisms) and differential root exudates in rhizosphere microecology of *Populus* in this ecological niche. The objectives of this study were to (i) identify the major factors influencing microbial communities; (ii) identify the key rhizosphere microorganisms in *Populus*; and (iii) examine the relationships between the exudates and rhizosphere microorganisms in the *Populus* microecosystem. The findings will lay a theoretical foundation for the screening and identification of key rhizosphere growth promoting microorganisms and the development and application of growth promoting agents for *Populus*.

## Materials and methods

### Study site and sample collection

The soil was sampled at the Research Base of Beijing Forestry University in Guanxian, Shandong Province, China (115°22′10.5″E, 36°30′56″N). The main cultivars of the experimental forests are *Populus tomentosa* (LM), *Populus nigra* (HY), *Populus alba* var. *pyramidalis* Bge. (XJ), and *Populus simonii* Carr (XY). Therefore, four *Populus* species (14 years old) were selected for study. They are widely distributed and cultivated in China and has been widely used in the numerous labs for diverse studies (Song et al., 2016, 2021; Ma et al., 2018; Sun et al., 2021). In this study, soil samples were randomly collected using the five-point sampling method with three replicates, five-point sampling (1 m × 1 m) was carried out using the diagonal principle. Plant fine roots (< 2 mm) were collected from each point within the root zone at a depth of 10–30 cm. The root samples were divided into two parts. For one part, the root-attached soil was shake off as bulk soils, and the soils tightly adhering to the roots were collected with a hairbrush as rhizosphere soil (Edwards et al., 2015; Zhu et al., 2022). Then, the soil sample was stored at 4°C until the physicochemical parameters were measured. For the other part, the samples were washed in 10 mM PBS buffer for 10 min on a shaking platform (120 rpm) and then transferred to clean 50 ml plastic tubes. Sterile tweezers were used to remove roots from the 50 ml plastic tubes, the soil particles directly centrifuged (6,000 × *g*, 4°C, 20 min) from the remaining suspension represented the rhizosphere samples (Beckers et al., 2016, 2017; Qin et al., 2018). And then the samples were stored at −20°C for later DNA extraction.

### Soil physicochemical parameters

The soil pH and the electric conductivity (EC) value were tested using a glass electrode meter in a suspension of 1 g of soil in 5 ml of distilled water by pH meter and conductivity meter. Moisture content was determined by the oven dry-weight method. Available phosphorus (AP), available potassium (AK), available nitrogen (AN), and organic matter (OM) were separately measured by the molybdenum blue, flame photometry, potassium persulfate oxidation, and dichromate oxidation method, respectively (Lu, 1999). Each measurement had three replicates. Furthermore, Tukey’s test was performed to evaluate the distribution of the soil physicochemical parameters. A *p*-value <0.05 was considered significant.

### Liquid chromatography with tandem mass spectrometry untargeted metabolomic analysis

After homogenization, a 1 g soil sample was put in a 5 ml Eppendorf tube, to which 3 ml of methanol was then added. The mixture was sonicated for 30 min in an ultrasonic bath. The supernatant was collected by centrifugation. These steps were repeated twice. Freeze-dried samples from exudates were resuspended in 150 μl methanol and then sonicated for 5 min in an ultrasonic bath. An aliquot of the supernatant was passed through a 0.22-μm (micropore) organic filter membrane and transferred to vials for subsequent analysis. The supernatant underwent high-performance liquid chromatography/triple time-of-flight mass spectrometry (Sciex TripleTOF 5600+, Framingham, MA, United States) analysis; an electron spray ionization (ESI) source was equipped. Separation was achieved on an HSS T3 column (100 mm × 2.1 mm, 1.7 μm; Waters, Milford, MA, United States). A gradient elution conditions were presented in Supplementary Table S1. The mass spectrometer was operated in both negative and positive ionization modes, and full scan mass spectra were recorded across the range of m/z 50–1,200. The curtain gas, gas 1, and gas 2 of the mass spectrometer were set to 35, 60, and 60 psi, respectively. The ESI temperature and spray voltage were 550°C and 4,500 V (negative)/5,500 V (positive), respectively. The differential metabolites with significant differences between any two of the four *Populus* samples were screened and identified by plant exudates databases (KEGG, PlantCyc, GMD; Wanichthanarak et al., 2020; Hawkins et al., 2021; Duan et al., 2022).

### DNA extraction and high-throughput sequencing

Total microbial genomic DNA was extracted from 0.1 g of soil sample using the DNeasy PowerSoil DNA Isolation kit (Qiagen, Hilden, Germany). The V3–V4 regions of the bacterial 16S rRNA genes for the entire bacterial communities were amplified using the 338F/806R primers and the following temperature cycling conditions described by literature (Song et al., 2020). Fungal internal transcribed spacer (ITS1) genes were amplified using the ITS1F and ITS2R barcode primers, with the amplification program according to the reference description (Zhou et al., 2021).

The purified PCR amplified products were paired-end sequenced (2 × 300) on the Illumina MiSeq platform (Illumina, San Diego, CA, United States). The raw data were first screened and sequences were removed from consideration if they were shorter than 120 bp, had a low quality score (≤20), contained ambiguous bases or did not exactly match to primer sequences and barcode tags, and separated using the sample-specific barcode sequences. Qualified reads were clustered into operational taxonomic units (OTUs) at a similarity level of 97% use Uparse algorithm of Vsearch (v2.7.1) software. The Ribosomal Database Project (RDP) Classifier tool was used to classify all sequences (16S rRNA genes) into different taxonomic groups against SILVA128 database. The BLAST tool was used to classify all sequences (ITS1 genes) into different taxonomic groups against Unite database. Sequences belonging to archaea, mitochondria, and chloroplasts were also removed. To avoid potential bias caused by differences in sequencing depth, the number of sequences in each sample was rarefied to 56,232 (16S rRNA genes) and 29,376 (ITS1 genes) sequences per sample (Zhou et al., 2022). The raw sequencing data were deposited in the Sequence Read Archive at NCBI with the accession number PRJNA888251.

### Statistical analysis

SPSS software (var. 17.0) was used for the data analysis. One-way ANOVA was used to detect differences among samples. Tukey’s test was performed to evaluate the distribution of the data. A value of *p* < 0.05 was considered significant. Metabolites with value of *p* < 0.05 and fold change >1.20 were considered as differential root exudates by SIMCA software (14.1), the fold change was compared with any two of four *Populus* species in pairs. Redundancy analysis (RDA) was performed using Canoco (v.5.0). A variation partitioning analysis (VPA) was applied to quantify the contributions of the environmental variables to the microbial communities, and the correlations between the microbes and metabolites, using R software 3.6.3. Interactions between microbial composition were studied through network analysis. The Gephi software 9.2 was used to visualize the network. The high values of topological features (degree, betweenness, and closeness centrality) suggest a core position of a node in the network (Jiao et al., 2017, 2020), and the high values of the above three parameters can jointly reflect the importance of key microbial flora (nodes in the network; Xing et al., 2021). Therefore, we choose high values of the topological parameters (degree, betweenness, and closeness centrality) as keystone taxa in this study.

The normalized stochasticity ratio (NST) was quantitated with the “NST” R package, which is assembly with 50% as the boundary point between more deterministic (NST < 50%) and more stochastic (NST > 50%) assembly (Ning et al., 2019). Levin’s niche breadth values of the dominant and keystone taxa were performed to illustrate community sensitivity to the environment, using the beta diversity “niche width” function within the R package “spaa” (Levins, 1968).

## Results

### Soil physicochemical characteristics and composition of root exudates

Most soil properties, including pH, moisture, OM, AN, AP, and AK, differed among the four *Populus* species (Table 1). The pH and EC values among HY, LM, XJ, and XY were 8.03–8.13 and 117.10–124.57 μs/cm, respectively. The moisture values of HY, LM, XJ, and XY were 2.00, 4.00, 4.00, and 3.00%, respectively. The average OM content was in the following order: LM (11.19 g/kg) > HY (9.98 g/kg) > XJ (7.74 g/kg) > XY (7.43 g/kg). The average AP contents of HY, LM, XJ, and XY were 4.34, 32.83, 11.50, and 2.90 mg/kg, respectively. The mean AN value were LM (38.93 mg/kg) > XJ (35.33 mg/kg) > HY (33.92 mg/kg) > XY (28.09 mg/kg). The AK values among HY, LM, XJ, and XY were 9.67, 10.50, 26.48, and 10.87 mg/kg, respectively. OM and AN content were significantly higher in LM than in the others. XJ had the highest AK content among the four *Populus* cultivars.

**Table 1:** Soil properties in the rhizosphere of four *Populus* species.

Soil properties	LM	XJ	HY	XY
pH	8.12 ± 0.04^a^	8.03 ± 0.03^b^	8.13 ± 0.02^a^	8.06 ± 0.03^ab^
Moisture	4.00% ± 0.00^a^	4.00% ± 0.00^a^	2.00% ± 0.00^b^	3.00% ± 0.00^a^
EC(μs/cm)	121.80 ± 0.70^a^	124.57 ± 0.74^a^	119.93 ± 0.32^a^	117.10 ± 6.90^a^
OM(g/kg)	11.19 ± 0.40^a^	7.74 ± 0.17^c^	9.98 ± 0.08^b^	7.43 ± 0.34^c^
AP(mg/kg)	32.83 ± 0.02^a^	11.50 ± 0.03^b^	4.34 ± 0.00^c^	2.90 ± 0.01^c^
AK(mg/kg)	10.50 ± 0.16^c^	26.48 ± 0.06^a^	9.67 ± 0.15^d^	10.87 ± 0.12^b^
AN(mg/kg)	38.93 ± 0.20^a^	35.33 ± 0.91^b^	33.92 ± 1.38^b^	28.09 ± 1.07^c^

Different lower letters mean significant differences (*p* < 0.05) between the properties of different samples. *Populus* species: LM (*P. tomentosa*), XJ (*P. alba* var. pyramidalis Bge.), HY (*P. nigra*), and XY (*P. simonii* Carr).

Thirty-nine differential metabolites with significant differences between any two of the four samples (LM, HY, XY, and XJ) were obtained (*p* < 0.05, fold change > 1.20). These metabolites predominantly consisted of fatty acyls, prenol lipids, carboxylic acids and derivatives, benzene and substituted derivatives, organooxygen compounds, and flavonoids. Seven plant root exudates were then identified from them, including coumarins (luvangetin), phenolic compounds (salicylic acid, gentisic acid, and oleuropein), a phytohormone (strigol), a flavonoid compound (chrysin), and fatty acids (linoleic acid; Table 2).

**Table 2:** The peak area of differential root exudates in the rhizosphere of four *Populus* species.

Differential root exudates	Mean value in each type of *Populus*				Pair-wise test (*P*-value)					
	LM	XJ	HY	XY	LM vs. XJ	LM vs. HY	LM vs. XY	XJ vs. HY	XJ vs. XY	HY vs. XY
Chrysin	0.000 ± 0.000	1.250 ± 0.170	29.348 ± 20.686	362.294 ± 176.755	0.041	0.028	0.001	0.045	0	0.001
Luvangetin	8463.262 ± 791.139	4171.554 ± 161.516	11513.344 ± 750.233	23666.135 ± 1231.016	0	0.001	0	0	0	0
Oleuropein	17.356 ± 5.709	435.355 ± 22.632	79.344 ± 5.573	104.369 ± 9.892	0	0	0	0	0	0.011
Salicylic acid	213.105 ± 11.562	275.985 ± 40.937	458.952 ± 38.372	336.125 ± 1.119	0.006	0	0	0	0.013	0.004
Strigol	261.810 ± 26.201	1.558 ± 0.404	7.565 ± 0.481	3.890 ± 0.782	0	0	0	0	0.01	0
Gentisic acid	59.848 ± 2.256	23.370 ± 3.041	155.988 ± 24.740	277.597 ± 77.996	0	0.001	0	0	0	0.02
Linoleic acid	77.894 ± 10.874	29.017 ± 0.545	21.263 ± 2.651	14.891 ± 2.451	0	0	0	0.004	0	0.019

*p* < 0.05, fold change > 1.20; *Populus* species: LM (*P. tomentosa*), XJ (*P. alba* var. pyramidalis Bge.), HY (*P. nigra*), and XY (*P. simonii* Carr).

XY had higher contents of chrysin, luvangetin, and gentisic acid compared to the other *Populus* cultivars, and the contents of luvangetin and gentisic acid in XY were 5.67 and 11.88 times higher than in XJ. Relatively high oleuropein content was detected in XJ. The salicylic acid content in HY was 1.37–2.15 times higher than that in LM, XJ, and XY. In this study, LM had the highest strigol content (approximately 168-fold change).

### Microbial diversity analysis

**Figure 1:**
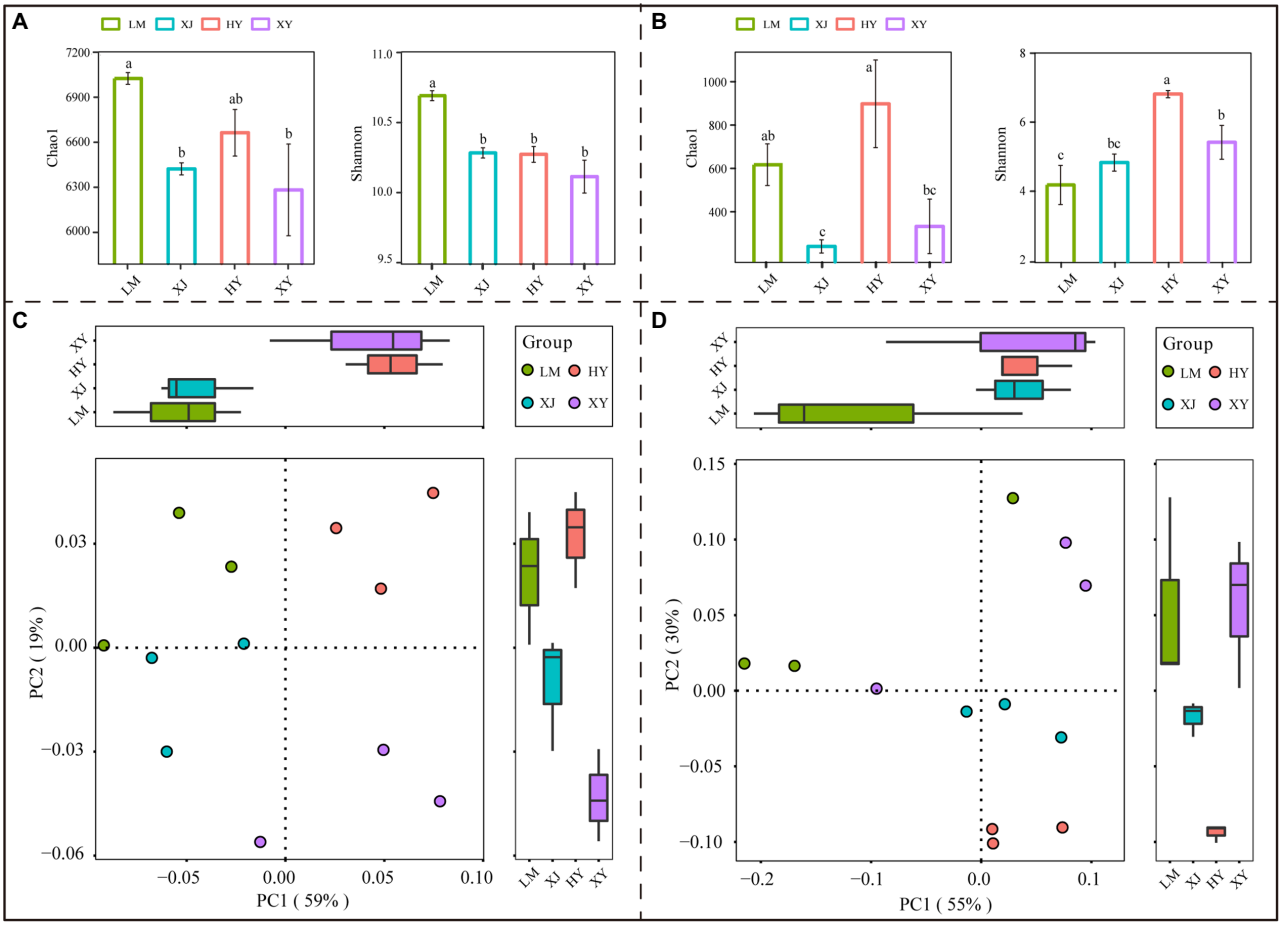
Chao1 index, Shannon index, and principal-coordinate analysis (PCoA) of bacteria and fungi from four *Populus* species. **(A)** The bacterial alpha diversity. **(B)** The fungal alpha diversity. **(C)** The bacterial principal component analysis. **(D)** The fungal principal component analysis. Different lower letters mean significant differences (*p* < 0.05). The same lowercase letters mean that the difference is not significant in four *Populus* species (*p* > 0.05).

The Chao1 and Shannon diversity indices were used to measure the microbial alpha-diversity of each soil sample; the highest bacterial Chao1 and Shannon indices were observed in the LM sample, followed by the HY and XJ samples; the XY sample had the lowest index values (Figure 1A), showing that the sum of bacterial species was the highest in the LM rhizosphere sample. Among the rhizosphere fungal community samples (Figure 1B), the HY cultivar harbored more diverse communities than the LM, XJ, and XY cultivars. Bray-Curtis analysis showed that bacterial and fungal diversity differed among the four *Populus* cultivars (Figures 1C,D).

### Microbial community composition

**Figure 2:**
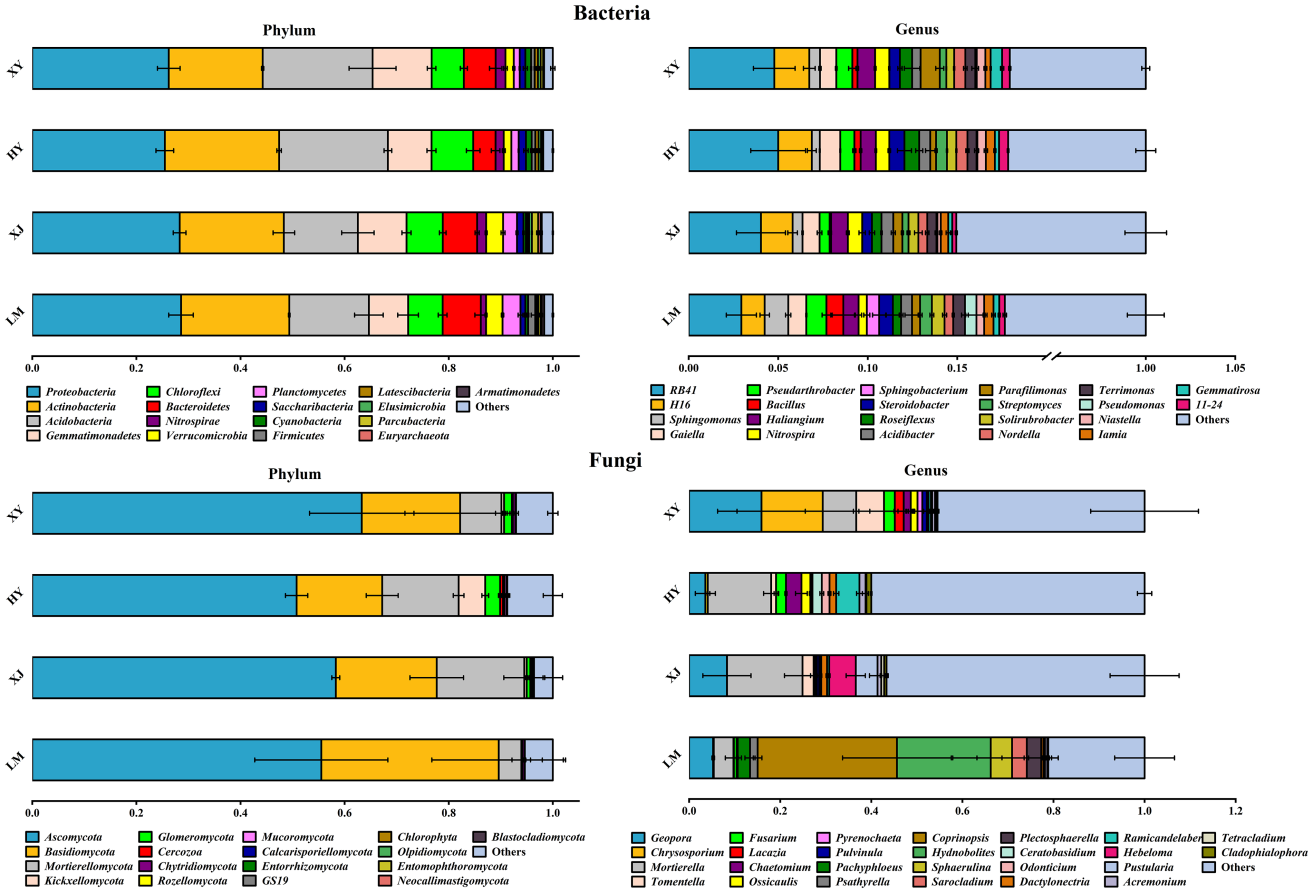
Rhizosphere microbial community compositions of four *Populus* species.

At the bacterial level, the communities were composed of the top 15 phyla and genera (dominant taxa) in each sample (Figure 2). Proteobacteria was the dominant phylum (25–28%), followed by Actinobacteria (18–21%), Acidobacteria (14–21%), Gemmatimonadetes (7–11%), Bacteroidetes (4–7%), and Chloroflexi (6–8%). At the genus level, soil beneficial bacteria included *RB41* and *H16*, with mean relative abundances of 2.9–4.9 and 1.3–1.9%, respectively, followed by *Sphingomonas, Gaiella, Haliangium*, and *Bacillus*.

At the fungal level, the communities were composed of the top 15 phyla and 10 genera (dominant taxa) in each sample (Figure 2). Ascomycota, Basidiomycota, and Mortierellomycota were the dominant fungal phyla, accounting for 50–63, 16–34, and 4–16%, respectively. At the genus level, the fungal communities were primarily composed of *Geopora*, *Chrysosporium*, *Mortierella*, and *Tomentella*. *Coprinopsis* was the most abundant fungal genus in LM. *Mortierella* dominated, with an abundance of 30–40% in HY and XY.

### Co-occurrence network of rhizosphere microorganisms

**Figure 3:**
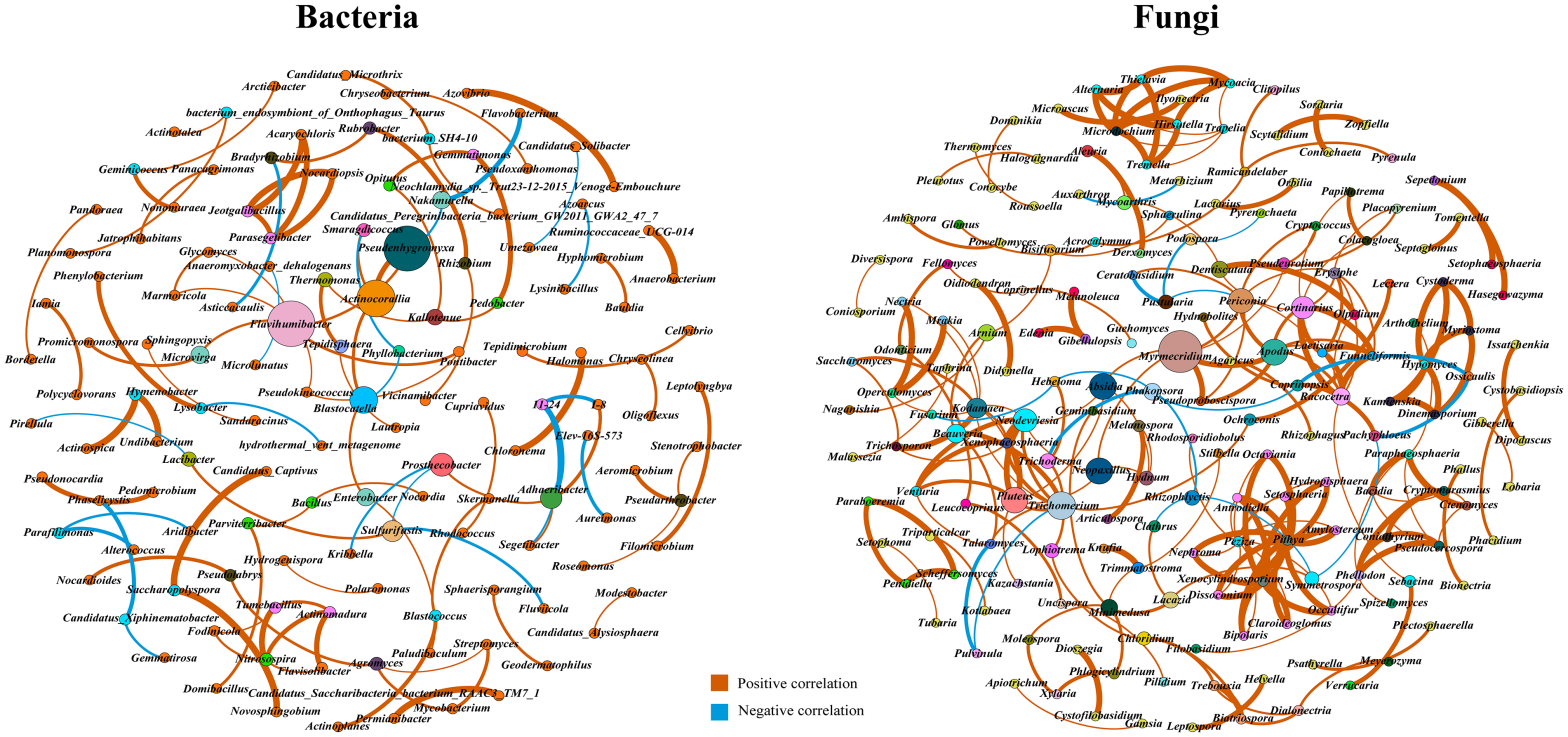
Co-occurrence networks of bacterial and fungal communities based on Spearman correlation.

The bacterial co-occurrence network composed of 132 nodes (genus) and 103 edges (links) was shown in Figure 3, which included 82 (79.61%) positive and 21 (20.39%) negative interactions. Positive relationships between microbial populations show the occurrence of a mutualistic interaction, while negative relationships indicate competition for hosts or a predacious relationship between microorganisms (Chow et al., 2014; Jiang J. et al., 2017). These interactions are strongly associated with important soil processes. The network had a modularity index of 0.94, the modularity index exceeded 0.4, suggesting a modular structure of the micro-network (Liu et al., 2020; Ye et al., 2020). Keystone taxa are the taxa which have major influence on microbiome composition and function at a particular space or time (Banerjee et al., 2018). The high values of the topological parameters (degree, betweenness, and closeness centrality) in the network were considered the keystone taxa (Jiao et al., 2017, 2020; Xing et al., 2021). The key bacteria were *Flavihumibacter*, *Blastocatella*, *Adhaeribacter*, *Microvirga*, *Agromyces*, and *Nitrosospira*. *Flavihumibacter* was positively correlated with *Microvirga*, indicating commensalism or a mutualistic relationship. The changes directly linked with the nodes (*Nitrosospira*) were all positive interactions.

The fungal co-occurrence network composed of 182 genera and 232 links was shown in Figure 3, which included 215 (92.67%) positive and 17 (7.33%) negative interactions. The network had a diameter of 13, average clustering coefficient of 0.33, average path length of 4.32, and modularity index of 0.88. The key microorganisms were mainly *Myrmecridium*, *Trichomerium*, *Absidia*, *Neopaxillus*, *Trichoderma, Pluteus*, *Periconia*, *Cortinarius*, *Beauveria*, *Talaromyces*, and *Arnium*. All of these taxa positively interacted with the *Beauveria* nodes. Eight genera, including *Apodus*, *Laetisaria*, *Racocetra*, *Funneliformis*, *Erysiphe*, *Dentiscutata*, *Colacogloea*, and *Papiliotrema*, were positively correlated with the keystone taxa *Cortinarius*, indicating their similarity or cooperation in ecological functions. There were two positive edges and one negative edge directly associated with *Trichoderma* hubs, including *Lophiotrema*, *Hebeloma*, and *Phakopsora*. *Trichoderma* was negatively correlated with *Fusarium* indirectly. Furthermore, key microorganisms generally have lower relative abundances than dominant microorganisms (Supplementary Table S1).

### Relationship between the microbial communities, soil properties, and differential root exudates

**Figure 4:**
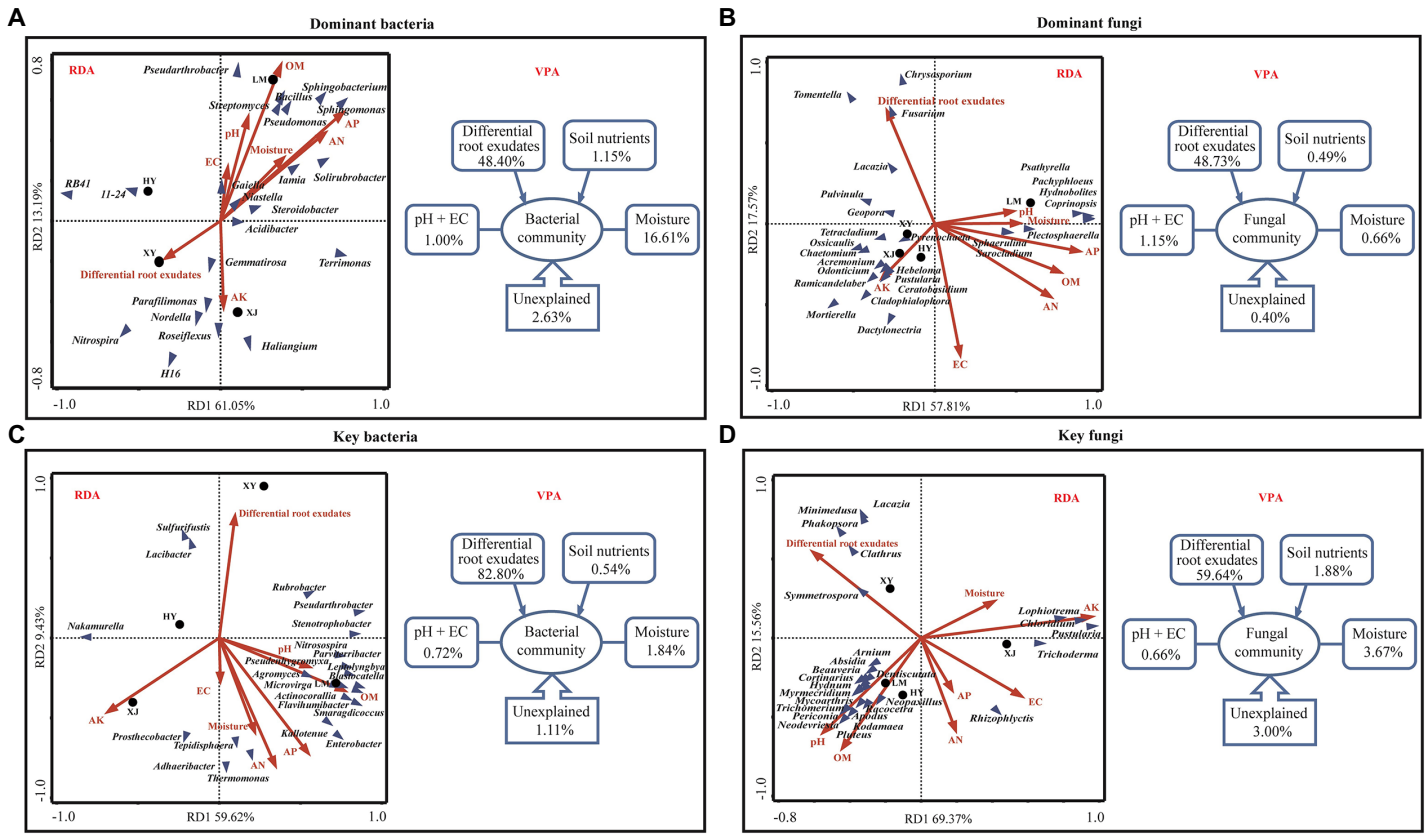
Redundancy analysis and variation partitioning analysis of microorganisms and soil properties. **(A)** Dominant bacteria and soil properties. **(B)** Dominant fungi and soil properties. **(C)** Key bacteria and soil properties. **(D)** Key fungi and soil properties. Red arrow: soil properties, blue triangle: microorganisms (In RDA).

Redundancy analysis and VPA were performed to study the relationships between the environmental factors and abundance of microbes (dominant taxa and keystone taxa). The RDA results shown in Figure 4A (dominant bacteria) indicated that OM, AN, AP, and moisture were positively correlated with the abundance of *Sphingomonas*, *Streptomyces*, *Bacillus*, and *Pseudomonas*, suggesting that bacteria were favored in rich nutritional conditions where they play important roles in soil C-, N- and P-cycling. AK and the differential root exudates negatively affected bacterial community composition. AP and AK had contrasting effects on the rhizosphere microbial population, indicating that they play a crucial role in the distribution of the microbial communities. The contribution of environmental factors was further quantified by VPA, which showed that differential root exudates explained 48.40%, soil nutrients explained 1.15%, pH and EC explained 1.00%, and moisture explained 16.61% of the dominant bacterial variance. The RDA results for dominant fungi (Figure 4B) showed that *Hebeloma* and *Mortierella* were positively correlated with AK, while the most dominant fungi had a negative association with soil nutrients (AP, AN, and OM) and differential root exudates, suggesting that fungi may be more suitable for oligotrophic environments. Differential root exudates had negatively correlation with most of the fungi (Figure 4B). VPA revealed that the differential root exudates explained 48.73% of the variance, soil nutrients explained 0.49%, pH and EC explained 1.15%, and moisture explained 0.66%.

The RDA results shown in Figures 4C,D indicated that OM and pH were positively correlated with the great majority of key microorganisms mainly include *Microvirga*, *Agromyces*, *Blastocatella*, *Apodus*, *Pluteus*, and *Trichomerium*, while the most key microorganisms had a negative association with AK and differential root exudates. VPA revealed that the differential root exudates explained 82.80% of the variance for key bacteria and 59.64% of the variance for key fungi, soil nutrients explained 0.54% (key bacteria) and 1.88% (key fungi), pH and EC explained 0.72% (key bacteria) and 0.66% (key fungi), and moisture explained 1.84% (key bacteria) and 3.67% (key fungi). Differential root exudates explained more of the variance in the bacterial and fungal communities than environmental factors, such as pH, EC, moisture, and soil nutrients. This finding indicates that the differential root exudates played the dominant role in shaping the *Populus* rhizosphere microbial communities and the differential root exudates had more effect on keystone taxa than dominant taxa.

### Correlations between the differential root exudates and microbial communities and microbial community assembly processes

**Figure 5:**
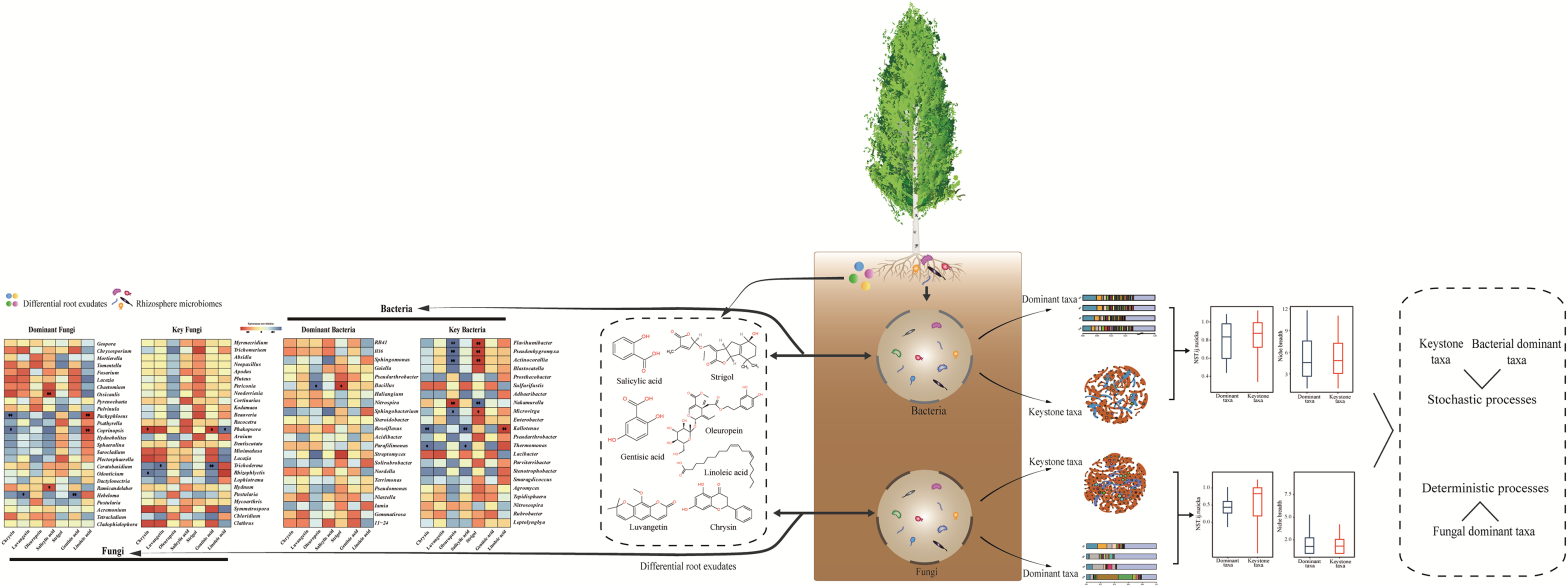
Diagram of microbial assembly process. Significance of each exudate: ^*^p ≤ 0.05. ^**^p ≤ 0.01.

Variation partitioning analysis showed that differential root exudates had largest contributions to the changes in the rhizosphere microbial communities (Figure 4), we further focused on analyzing the relationships between differential root exudates and microbes (dominant and key microorganisms) in the rhizosphere by Spearman’s correlation analysis. The correlation analysis revealed that strigol was positively correlated with the dominant bacteria *Bacillus* (Figure 5). Phenolic compounds (oleuropein, gentisic acid, and salicylic acid) were negatively correlated with two dominant taxa (*Bacillus* and *Hebeloma*) and positively associated with two genera (*Ossicaulis* and *Ramicandelaber*). Luvangetin showed negatively relationship with *Hebeloma*. The abundances of dominant fungi (*Pachyphloeus* and *Coprinopsis*) had negatively relationship with chrysin and positively relationship with linoleic acid.

The correlation analysis indicated that strigol positively correlated with the abundances of the key bacteria *Microviga*, *Pseudenhygromyxa*, *Flavihumibacter*, and *Actinocorallia*. Oleuropein and other phenolic compounds had significantly negative correlation with the key microorganisms. Negative correlations were detected between the phenolics and abundances of bacteria (*Flavihumibacter*, *Pseudenhygromyxa*, *Actinocorallia*, *Microviga*, *Kallotenue,* and *Thermomonas*) and fungi (*Trichoderma*). Gentisic acid (a phenolic compound) was negatively correlated with *Trichoderma*. Chrysin (flavonoid compounds) was negatively correlated with three keystone taxa (*Kallotenue*, *Thermomonas,* and *Rhizophlyctis*). *Kallotenue* was positively associated with linoleic acid. In general, strigol was positively correlated with key bacteria, whereas phenolic compounds showed a negative correlation, which indicated the response of keystone taxa and dominant taxa were different to the same variables.

To study more in depth the differences in community structure, we further calculated the community-level habitat niche breadths (Bcom). Bacterial keystone taxa exhibited higher Bcom value than dominant taxa, fungal dominant taxa showed higher Bcom value than keystone taxa. Subsequently, we further employed NST to elucidate the assembly processes. The NST value was above the 50% boundary point accounted for both dominant taxa and keystone taxa (for 90.91%), suggesting that stochastic process played a more important role than deterministic process during the bacterial assembly. Additionally, NST value was observed in bacterial keystone communities with an average of 81.69% than that in bacterial dominant communities with an average of 79.43%. The dominant fungi accounted for 42.43% and key fungi accounted for 68.18% in the NST values above 50%, implying that fungal dominant taxa were dominated by deterministic process.

## Discussion

### Rhizosphere characteristics of *Populus*

Many studies have suggested that soil properties are the basic factors affecting the soil microbial communities (Li X. et al., 2021). The basic metabolism of microorganisms depends strongly on soil moisture (Kundel et al., 2020; Su et al., 2020). Soil OM is the main substrate and energy source for soil microbes (Wang J. et al., 2021). A previous report showed that AK alters the microbial communities and improves soil metabolic activities and functional diversity (Li H. et al., 2021). In the present study, we found that LM and XJ had high trophic level soils (Table 1).

Moreover, root exudates also affect the formation and structure of rhizosphere microbiome (Czarnota et al., 2003; Vives-Peris et al., 2020). Here we found seven differential root exudates. Oleuropein and gentisic acid are phenolic compounds, and gentisic acid is one of the most common aromatic acids in plants (Zhang et al., 2017; Catinella et al., 2022). Salicylic acid content can vary with genotype and the developmental stage of the plants (Veach et al., 2019). Salicylic acid regulates plant growth and development that as a common plant phenolic compound in Salicaceae, and acts as an inducible defense chemical expressed in response to a pathogen. However, some researches have showed that salicylic acid reduces soil microbial diversity and significantly affects the carbon metabolism capacity and genetic structure of microbial communities. Strigol is a carotenoid-derived signaling molecule that communicates with parasites in the rhizosphere, and regulates plant growth and development by crosstalk with other hormones (Wakabayashi et al., 2021). Strigol is chemically unstable and decomposes rapidly in the soil, but exudates can protect strigol from rapid degradation (Yoneyama, 2020). The linoleic acid level was higher in LM than in the others significantly. Linoleic acid is a major component of biomembranes; it functions as a signaling molecule and directly participates in development (Huang et al., 2021). The changes of some specific compounds in root exudates can affect the dynamics of soil microbial communities (Zhao et al., 2021).

### Dominant and keystone taxa on *Populus* rhizosphere

We revealed that the dominant phyla such as Proteobacteria, Actinobacteria, Acidobacteria, Ascomycota, and Basidiomycota. The ratio of Proteobacteria to Acidobacteria can be used to measure the level of soil nutrition, favoring Proteobacteria in rich soils and Acidobacteria in poor soils (Castro et al., 2010; Gottel et al., 2011). In our research, LM and XJ had higher ratios than HY and XY. Bacteroidetes is usually not a dominant bacterial phylum in soil (Song et al., 2020), but was highly abundant in the rhizosphere. *Sphingomonas*, a Gram-negative bacterium and rhizosphere biomarker that grows under aerobic conditions. The biomarker results indicated that the *Populus* rhizosphere environment can provide sufficient air and organic nutrient conditions for these bacteria (Li J. et al., 2020). *Mortierella* is a plant growth-promoting fungi that enhances plant phosphate nutrition (Li F. et al., 2020). *Geopora* species are common and important ectomycorrhizal fungi that often seen in woody plants rhizosphere, where they can increase the utilization of water and nutrients by hosts. These taxa constituted a high proportion of the microbial communities in the different *Populus* cultivars, and play a notable role in promoting plant growth and degrading refractory pollutants, and protect plants from harmful pathogens (Patterson et al., 2019).

Dominant and key populations affect the composition and structure of the entire microbial communities (Berry and Widder, 2014). The entire communities will crumble without key species (Floc’H et al., 2020); thus, apart from those dominant taxa, identifying the keystone taxa of the plant rhizosphere is essential to optimize plant growth. Modularity, which represents microbial interactions, is vital for microbial community stability and resilience due to the resource allocation, habitat heterogeneity, phylogeny, or niche overlap (Tya et al., 2021). In this research, the modularity index exceeded 0.4, suggesting a modular structure of the real-world network (Liu et al., 2020; Ye et al., 2020). The co-occurrence network composed of 22 bacterial keystone taxa and 26 fungal keystone taxa. The genus *Microvirga* is very common in nature. *Microvirga* sp. participates in the soil nitrogen cycle and provides nitrogen for plants and other microorganisms (Amin et al., 2016; Veyisoglu et al., 2016; Longa et al., 2017). The key genus *Adhaeribacter* exhibits urease and alkaline phosphomonoesterase activities, which help maintain available N and P content in the soil and regulate growth in environments polluted with heavy metals (Lin et al., 2020). *Nitrosospira* are ammonia- and nitrite-oxidizing bacteria, related to the nitrogen metabolic processes that make nutrients available to other community members. Numerous studies have revealed that nitrogen cycling taxa play a key role in a variety of ecosystems (Rädecker et al., 2015; Che et al., 2018; Dai et al., 2020; Ramond et al., 2022). Therefore, the ecological role and mechanism of nitrogen cycling-related microorganisms in plant growth merit further systematic study.

*Beauveria bassiana* is widely used as an effective strain for biological control of plant diseases. The BbAFP1 chitinase secreted by *B. bassiana* has good antifungal activity in plants, and its gene expression in Chinese white poplar can well reduce the incidence of diseases caused by *Cytospora chrysosperma* (Tong et al., 2021). Members of the ectomycorrhizal genus *Cortinarius* are often dominant in fungal communities from boreal soils. Some members of this genus may be directly involved in the degradation of soil OM in the humus layers of northern forest ecosystems. This is an active mycorrhizal community with high mycelial turnover rates that successively depletes N humus and minimizes the long-term accumulation of humus layers, which were very important in maintaining the balance of the ecosystem (Bödeker et al., 2014; Clemmensen et al., 2015). The genus *Trichoderma*, a well-known plant growth-promoting microorganism and biological control agent (Fernández-González et al., 2020), activates the plant immune system through induced systemic resistance, which is a priming defense mechanism against pathogens (Gupta et al., 2021). Xylanase secreted by *Trichoderma* promotes systemic resistance of host plants against pathogens, suggesting that *Trichoderma* may have biocontrol abilities in the *Populus* rhizosphere (Guo et al., 2021). Furthermore, key microorganisms generally have lower relative abundances than dominant microorganisms, indicating that species interactions may played a more important role in *Populus* growth than that of species abundance.

### Effects of differential root exudates on rhizosphere microbial communities

As shown in RDA, environmental factors such as AN, OM, and pH influence microbial communities. Here, we found by VPA that the differential root exudates explained more of the variability in shaping the *Populus* rhizosphere microbiome than other environmental factors. This result was in agreement with previous reports that root exudates significantly regulate the soil microbial community structure (Gu et al., 2020). We further revealed that the correlations of the differential root exudates and microbiome (dominant taxa and keystone taxa). Our results showed that strigol and phenolic compounds were key factors for rhizosphere microbial communities. Strigol was reported to regulate root architecture and hyphal branching of AMF (Liu et al., 2018). *Bacillus* plays an important mediating role in mycorrhizal symbiosis, which improves the growth and nutrient uptake of plants (Wang Y. et al., 2021; Zhang et al., 2021). Therefore, the positive correlation between strigol and *Bacillus* may have combined action on the development of mycorrhiza, which could promote the growth of *Populus*. Phenolic compounds showed a negative correlation with microbiome, probably because these chemicals are relatively resistant to decomposition by microorganisms and inhibit microbial activity (Gu et al., 2020). Furthermore, phenolic compounds can impair membrane structure and function, or increase the permeability of cell membrane to cause the leakage of cell contents (Mattos et al., 2017). It is reported that salicylic acid modulates root colonization by *Trichoderma*, as exogenous inoculation decreases the multiplication of the fungus on roots. Phenolic compounds extracted from rice straw were also reported to have significant inhibition on the growth of *T. reesei* (Zheng et al., 2017; Macías-Rodríguez et al., 2020). Moreover, the differential root exudates affected the key bacteria more than fungi and the dominant bacteria, and that the keystone taxa maintained the microbial ecology critically. Therefore, the further study on the relationship between specific microorganisms and rhizosphere exudates will provide a theoretical basis for reducing the adverse effects and promoting plant growth.

### Differences in community assembly between keystone and dominant taxa

In the present study, we demonstrated that the niche-based stochastic processes determine the assembly of bacterial communities and fungal keystone taxa. Keystone taxa showed higher stochasticity comared dominant taxa, thus demonstrating keystone taxa make more contributions to the stable of community structure under environmental disturbances. In addition, a finding is that the NST value of bacteria was higher than that of fungi, suggesting that stochastic processes contribute more to bacterial communities than to fungal communities. This observation could be due to the size-plasticity hypothesis that smaller organisms (bacteria) are less environment filtered than larger organisms (Liu et al., 2015). Subsequently, we quantified the Bcom value. Bacterial keystone taxa had a higher Bcom value than dominant, which was consistent with previous studies, a previous study reported that organisms with Bcom value might have greater metabolic plasticity and be less influenced by deterministic processes (Pandit et al., 2009). The Bcom value of fungal keystone taxa was lower than that of dominant.

In a word, VPA indicated that the differential root exudates explained a larger proportion of keystone taxa (82.8 and 59.64%) than dominant taxa (48.4 and 48.73%), while NST demonstrated keystone taxa showed higher stochasticity compared dominant taxa. These contrasting results may be due to the unmeasured environmental variables as mentioned above. Thus, more comprehensive data (taxonomic, phylogenetic, environmental, and spatial) and more dimensions should be considered when relating the relative influence of ecological processes to microbial community assembly.

## Conclusion

In this study, differential root exudates from four *Populus* cultivars, including luvangetin, salicylic acid, gentisic acid, oleuropein, strigol, chrysin, and linoleic acid, were the major interfering factor of microbial communities’ variation. Network analysis revealed that keystone taxa interactions were very important in maintaining the stability of the *Populus* rhizosphere microorganisms than species abundance. Furthermore, correlation analysis revealed that strigol had positively effects with bacteria, the phenolic compounds and chrysin were negatively correlated with the rhizosphere microorganisms. In general, differential root exudates showed significant relationship with the key bacterial taxa. Community structure (keystone taxa and bacterial dominant taxa) was mostly determined by stochastic processes, keystone taxa showed higher stochasticity compared dominant taxa, thus demonstrating keystone taxa make more contributions to the stable of community structure under environmental disturbances. The results provided a basis for further identification and utilization of important microbes in *Populus* rhizosphere.

## Data availability statement

The data presented in the study are deposited in the Sequence Read Archive repository, accession number PRJNA888251, https://www.ncbi.nlm.nih.gov/bioproject/PRJNA888251.

## Author contributions

ML and ZS: conceptualization, methodology, investigation, writing—original draft, and writing—review and editing. ZL, PZ, and JX: software and formal analysis. RQ: validation and resources. CD: validation and data curation. HG: conceptualization, methodology, writing—review and editing, supervision, and funding acquisition. All authors contributed to the article and approved the submitted version.

## Funding

This work was funded by National Key Research and Development Program of China (2021YFD2201205), National Natural Science Foundation of China (41601513 and 41977203), and Beijing Forestry University Municipal Training Program of Innovation and Entrepreneurship for Undergraduates (202110022017).

## Conflict of interest

The authors declare that the research was conducted in the absence of any commercial or financial relationships that could be construed as a potential conflict of interest.

## Publisher’s note

All claims expressed in this article are solely those of the authors and do not necessarily represent those of their affiliated organizations, or those of the publisher, the editors and the reviewers. Any product that may be evaluated in this article, or claim that may be made by its manufacturer, is not guaranteed or endorsed by the publisher.

## Supplementary material

The Supplementary material for this article can be found online at: https://www.frontiersin.org/articles/10.3389/fmicb.2022.1042944/full#supplementary-material

## References

[ref2] Amin A. Ahmed I. Habib N. Abbas S. Hasan F. Xiao M.. (2016). *Microvirga pakistanensis* sp. nov., a novel bacterium isolated from desert soil of Cholistan. Pak. Arch. Microbiol. 198, 933–939. doi: 10.1007/s00203-016-1251-3, PMID: 27290649

[ref3] Bahram M. Hildebrand F. Forslund S. K. Anderson J. L. Soudzilovskaia N. A. Bodegom P. M.. (2018). Structure and function of the global topsoil microbiome. Nature 560, 233–237. doi: 10.1038/s41586-018-0386-6, PMID: 30069051

[ref4] Bai Y. Ren P. Feng P. Yan H. Li W. (2020). Shift in rhizospheric and endophytic bacterial communities of tomato caused by salinity and grafting. Sci. Total Environ. 734:139388. doi: 10.1016/j.scitotenv.2020.139388, PMID: 32470659

[ref601] Banerjee S. Schlaeppi K. Van D. (2018). Keystone taxa as drivers of microbiome structure and functioning. Nat. Rev. Microbiol. 16, 567–576. doi: 10.1038/s41579-018-0024-1 29789680

[ref5] Beckers B. Op De Beeck M. Weyens N. Boerjan W. Vangronsveld J. (2017). Structural variability and niche differentiation in the rhizosphere and endosphere bacterial microbiome of field-grown poplar trees. Microbiome 5:25. doi: 10.1186/s40168-017-0241-2, PMID: 28231859 PMC5324219

[ref6] Beckers B. Op De Beeck M. Weyens N. Van Acker R. Van Montagu M. Boerjan W.. (2016). Lignin engineering in field-grown poplar trees affects the endosphere bacterial microbiome. Proc. Natl. Acad. Sci. 113, 2312–2317. doi: 10.1073/pnas.1523264113, PMID: 26755604 PMC4776533

[ref7] Berry D. Widder S. (2014). Deciphering microbial interactions and detecting keystone species with co-occurrence networks. Front. Microbiol. 5:219. doi: 10.3389/fmicb.2014.00219 24904535 PMC4033041

[ref8] Bödeker I. T. M. Clemmensen K. E. Boer W. Martin F. Olson Å. Lindahl B. D. (2014). Ectomycorrhizal *Cortinarius* species participate in enzymatic oxidation of humus in northern forest ecosystems. New Phytol. 203, 245–256. doi: 10.1111/nph.12791, PMID: 24725281

[ref9] Carrión V. J. Perez-Jaramillo J. Cordovez V. Tracanna V. de Hollander M. Ruiz-Buck D.. (2019). Pathogen-induced activation of disease-suppressive functions in the endophytic root microbiome. Science 366, 606–612. doi: 10.1126/science.aaw9285, PMID: 31672892

[ref10] Castro H. F. Classen A. T. Austin E. E. Norby R. J. Schadt C. W. (2010). Soil microbial community responses to multiple experimental climate change drivers. Appl. Environ. Microbiol. 76, 999–1007. doi: 10.1128/AEM.02874-09, PMID: 20023089 PMC2820983

[ref11] Catinella G. Donzella S. Borgonovo G. Dallavalle S. Contente M. L. Pinto A. (2022). Efficient 2-step enzymatic cascade for the bioconversion of oleuropein into hydroxytyrosol. Antioxidants 11:260. doi: 10.3390/antiox11020260, PMID: 35204142 PMC8868057

[ref12] Che R. Qin J. Tahmasbian I. Wang F. Zhou S. Xu Z.. (2018). Litter amendment rather than phosphorus can dramatically change inorganic nitrogen pools in a degraded grassland soil by affecting nitrogen-cycling microbes. Soil Biol. Biochem. 120, 145–152. doi: 10.1016/j.soilbio.2018.02.006

[ref13] Chow C.-E. T. Kim D. Y. Sachdeva R. Caron D. A. Fuhrman J. A. (2014). Top-down controls on bacterial community structure: microbial network analysis of bacteria, T4-like viruses and protists. ISME J. 8, 816–829. doi: 10.1038/ismej.2013.199, PMID: 24196323 PMC3960540

[ref602] Clemmensen K. E. Finlay R. D. Dahlberg A. Stenlid J. Wardle D. A. Lindahl B. D. (2015). Carbon sequestration is related to mycorrhizal fungal community shifts during long‐term succession in boreal forests. New Phytol. 205, 1525–1536. doi: 10.1111/nph.13208, PMID: 25494880

[ref14] Czarnota M. A. Rimando A. M. Weston L. A. (2003). Evaluation of root exudates of seven sorghum accessions. J. Chem. Ecol. 29, 2073–2083. doi: 10.1023/A:1025634402071 14584676

[ref15] Dai Z. Yu M. Chen H. Zhao H. Huang Y. Su W.. (2020). Elevated temperature shifts soil N cycling from microbial immobilization to enhanced mineralization, nitrification and denitrification across global terrestrial ecosystems. Glob. Chang. Biol. 26, 5267–5276. doi: 10.1111/gcb.15211, PMID: 32614503

[ref16] Duan M. Lu J. Yang W. Lu M. Wang J. Li S.. (2022). Metabarcoding and metabolome analyses reveal mechanisms of *Leymus chinensis* growth promotion by fairy ring of *Leucocalocybe mongolica*. J. Fungi. 8:944. doi: 10.3390/jof8090944, PMID: 36135669 PMC9505569

[ref17] Edwards J. Johnson C. Santos-Medellín C. Lurie E. Podishetty N. K. Bhatnagar S.. (2015). Structure, variation, and assembly of the root-associated microbiomes of rice. Proc. Natl. Acad. Sci. 112, E911–E920. doi: 10.1073/pnas.1414592112, PMID: 25605935 PMC4345613

[ref18] Feng J. Jiang D. Shang H. Dong M. Wang G. He X.. (2013). Barcoding poplars (*Populus L*.) from Western China. PLoS One 8:e71710. doi: 10.1371/journal.pone.0071710, PMID: 23977122 PMC3747233

[ref19] Fernández-González A. J. Cardoni M. Gómez-Lama Cabanás C. Valverde-Corredor A. Villadas P. J. Fernández-López M.. (2020). Linking belowground microbial network changes to different tolerance level towards *Verticillium* wilt of olive. Microbiome 8:11. doi: 10.1186/s40168-020-0787-2, PMID: 32007096 PMC6995654

[ref20] Floc’H J. B. Hamel C. Harker K. N. St-Arnaud M. (2020). Fungal communities of the canola rhizosphere: keystone species and substantial between-year variation of the rhizosphere microbiome. Microb. Ecol. 80, 762–777. doi: 10.1007/s00248-019-01475-8 31897569

[ref21] Gottel N. R. Castro H. F. Kerley M. Yang Z. Pelletier D. A. Podar M.. (2011). Distinct microbial communities within the endosphere and rhizosphere of *Populus deltoides* roots across contrasting soil types. Appl. Environ. Microbiol. 77, 5934–5944. doi: 10.1128/AEM.05255-11, PMID: 21764952 PMC3165402

[ref22] Gu Y. Wang X. Yang T. Friman V. Geisen S. Wei Z.. (2020). Chemical structure predicts the effect of plant-derived low-molecular weight compounds on soil microbiome structure and pathogen suppression. Funct. Ecol. 34, 2158–2169. doi: 10.1111/1365-2435.13624

[ref23] Guo R. Ji S. Wang Z. Zhang H. Wang Y. Liu Z. (2021). *Trichoderma asperellum* xylanases promote growth and induce resistance in poplar. Microbiol. Res. 248:126767. doi: 10.1016/j.micres.2021.126767, PMID: 33873138

[ref24] Gupta R. Keppanan R. Leibman-Markus M. Rav David D. Elad Y. Ment D.. (2021). The entomopathogenic fungi *Metarhizium brunneum* and *Beauveria bassiana* promote systemic immunity and confer resistance to a broad range of pests and pathogens in tomato. Phytopathology 112, 784–793. doi: 10.1094/PHYTO-08-21-0343-R, PMID: 34636647

[ref25] Hawkins C. Ginzburg D. Zhao K. Dwyer W. Xue B. Xu A.. (2021). Plant metabolic network 15: a resource of genome-wide metabolism databases for 126 plants and algae. J. Integr. Plant Biol. 63, 1888–1905. doi: 10.1111/jipb.13163, PMID: 34403192

[ref26] Hogan J. A. Baraloto C. Ficken C. Clark M. D. Weston D. J. Warren J. M. (2021). The physiological acclimation and growth response of *POPULUS TRICHOCARPA* to warming. Physiol. Plant. 173, 1008–1029. doi: 10.1111/ppl.13498, PMID: 34272872

[ref27] Huang K. L. Tian J. Wang H. Fu Y.-F. Li Y. Zheng Y.. (2021). Fatty acid export protein BnFAX6 functions in lipid synthesis and axillary bud growth in *Brassica napus*. Plant Physiol. 186, 2064–2077. doi: 10.1093/plphys/kiab229, PMID: 34618109 PMC8331132

[ref28] Jiang Y. Li S. Li R. Zhang J. Liu Y. Lv L.. (2017). Plant cultivars imprint the rhizosphere bacterial community composition and association networks. Soil Biol. Biochem. 109, 145–155. doi: 10.1016/j.soilbio.2017.02.010

[ref29] Jiang J. Song Z. Yang X. Mao Z. Nie X. Guo H.. (2017). Microbial community analysis of apple rhizosphere around Bohai gulf. Sci. Rep. 7:8918. doi: 10.1038/s41598-017-08398-9, PMID: 28827532 PMC5566992

[ref30] Jiao S. Chen W. Wei G. (2017). Biogeography and ecological diversity patterns of rare and abundant bacteria in oil-contaminated soils. Mol. Ecol. 26, 5305–5317. doi: 10.1111/mec.14218, PMID: 28665016

[ref31] Jiao S. Yang Y. Xu Y. Zhang J. Lu Y. (2020). Balance between community assembly processes mediates species coexistence in agricultural soil microbiomes across eastern China. ISME J. 14, 202–216. doi: 10.1038/s41396-019-0522-9, PMID: 31611655 PMC6908645

[ref32] Kundel D. Bodenhausen N. Jørgensen H. B. Truu J. Birkhofer K. Hedlund K.. (2020). Effects of simulated drought on biological soil quality, microbial diversity and yields under long-term conventional and organic agriculture. FEMS Microbiol. Ecol. 96:fiaa205. doi: 10.1093/femsec/fiaa205, PMID: 33016314 PMC7705324

[ref33] Levins R. (1968). Evolution in Changing Environments: Some Theoretical Explorations. Princeton, NJ: Princeton University Press.

[ref34] Li X. Chen J. Zhang Q. Li X. F. Zhou X. Tao Y. (2021). Microbial community responses to multiple soil disinfestation change drivers. Appl. Microbiol. Biotechnol. 105, 6993–7007. doi: 10.1007/s00253-021-11528-z, PMID: 34453565

[ref35] Li J. Luo Z. Zhang C. Qu X. Chen M. Song T.. (2020). Seasonal variation in the rhizosphere and non-rhizosphere microbial community structures and functions of *camellia yuhsienensis* Hu. Microorganisms 8:1385. doi: 10.3390/microorganisms8091385, PMID: 32927703 PMC7564921

[ref36] Li H. Qiu Y. Yao T. Han D. Gao Y. Zhang J.. (2021). Nutrients available in the soil regulate the changes of soil microbial community alongside degradation of alpine meadows in the northeast of the Qinghai-Tibet plateau. Sci. Total Environ. 792:148363. doi: 10.1016/j.scitotenv.2021.148363, PMID: 34465051

[ref37] Li F. Zhang S. Wang Y. Li Y. Li P. Chen L.. (2020). Rare fungus, *Mortierella capitata*, promotes crop growth by stimulating primary metabolisms related genes and reshaping rhizosphere bacterial community. Soil Biol. Biochem. 151:108017. doi: 10.1016/j.soilbio.2020.108017

[ref38] Lin H. Liu C. Li B. Dong Y. (2020). *Trifolium repens* L. regulated phytoremediation of heavy metal contaminated soil by promoting soil enzyme activities and beneficial rhizosphere associated microorganisms. J. Hazard. Mater. 402:123829. doi: 10.1016/j.jhazmat.2020.123829, PMID: 33254810

[ref39] Liu K. Ding X. Wang J. (2020). Soil metabolome correlates with bacterial diversity and co-occurrence patterns in root-associated soils on the Tibetan plateau. Sci. Total Environ. 735:139572. doi: 10.1016/j.scitotenv.2020.139572, PMID: 32480142

[ref40] Liu G. Pfeifer J. de Brito Francisco R. Emonet A. Stirnemann M. Gübeli C.. (2018). Changes in the allocation of endogenous strigolactone improve plant biomass production on phosphate-poor soils. New Phytol. 217, 784–798. doi: 10.1111/nph.14847, PMID: 29083039 PMC5765447

[ref41] Liu J. Sui Y. Yu Z. Shi Y. Chu H. Jin J.. (2015). Soil carbon content drives the biogeographical distribution of fungal communities in the black soil zone of Northeast China. Soil Biol. Biochem. 83, 29–39. doi: 10.1016/j.soilbio.2015.01.009

[ref42] Longa C. Nicola L. Antonielli L. Mescalchin E. Zanzotti R. Turco E.. (2017). Soil microbiota respond to green manure in organic vineyards. J. Appl. Microbiol. 123, 1547–1560. doi: 10.1111/jam.13606, PMID: 28990280

[ref43] Lu R.K. (1999). Soil and Agro-Chemistry Analytical Methods. Beijing: China Agricultural Science & Technology Press.

[ref44] Lu T. Ke M. Lavoie M. Jin Y. Fan X. Zhang Z.. (2018). Rhizosphere microorganisms can influence the timing of plant flowering. Microbiome 6:231. doi: 10.1186/s40168-018-0615-0, PMID: 30587246 PMC6307273

[ref610] Ma J. Wan D. Duan B. Bai X. Bai Q. Chen N.. (2018). Genome sequence and genetic transformation of a widely distributed and cultivated poplar. Plant Biotechnol. J. 17, 451–460. doi: 10.1111/pbi.12989, PMID: 30044051 PMC6335071

[ref45] Ma L. Yang L. Liu W. Zhang Y. Zhou Q. Wu Z.. (2021). Effects of root exudates on rhizosphere bacteria and nutrient removal in pond-ditch circulation systems (PDCSs) for rural wastewater treatment. Sci. Total Environ. 785:147282. doi: 10.1016/j.scitotenv.2021.147282, PMID: 33933761

[ref46] Macías-Rodríguez L. Contreras-Cornejo H. A. Adame-Garnica S. G. Del-Val E. Larsen J. (2020). The interactions of *Trichoderma* at multiple trophic levels: inter-kingdom communication. Microbiol. Res. 240:126552. doi: 10.1016/j.micres.2020.126552, PMID: 32659716

[ref47] Mattos G. N. Tonon R. V. Furtado A. A. Cabral L. M. (2017). Grape by-product extracts against microbial proliferation and lipid oxidation: a review: grape by-products with antimicrobial and antioxidant potential. J. Sci. Food Agric. 97, 1055–1064. doi: 10.1002/jsfa.8062, PMID: 27696415

[ref48] Min K. Freeman C. Kang H. Choi S.-U. (2015). The regulation by phenolic compounds of soil organic matter dynamics under a changing environment. Biomed. Res. Int. 2015, 1–11. doi: 10.1155/2015/825098, PMID: 26495314 PMC4606107

[ref49] Ning D. Deng Y. Tiedje J. M. Zhou J. (2019). A general framework for quantitatively assessing ecological stochasticity. Proc. Natl. Acad. Sci. 116, 16892–16898. doi: 10.1073/pnas.1904623116, PMID: 31391302 PMC6708315

[ref50] Pandit S. N. Kolasa J. Cottenie K. (2009). Contrasts between habitat generalists and specialists: an empirical extension to the basic metacommunity framework. Ecology 90, 2253–2262. doi: 10.1890/08-0851.1, PMID: 19739387

[ref51] Patterson A. Flores-Rentería L. Whipple A. Whitham T. Gehring C. (2019). Common garden experiments disentangle plant genetic and environmental contributions to ectomycorrhizal fungal community structure. New Phytol. 221, 493–502. doi: 10.1111/nph.15352, PMID: 30009496

[ref52] Qin Y. Pan X. Y. Jin W. Chen L. Q. Yuan Z. L. (2018). Comparison of four extraction methods of soil microbiome in poplar plantation. Sci. Silvae Sin. 9, 169–176. doi: 10.11707/j.1001-7488.20180919

[ref53] Rädecker N. Pogoreutz C. Voolstra C. R. Wiedenmann J. Wild C. (2015). Nitrogen cycling in corals: the key to understanding holobiont functioning. Trends Microbiol. 23, 490–497. doi: 10.1016/j.tim.2015.03.008, PMID: 25868684

[ref54] Ramond J. B. Jordaan K. Díez B. Heinzelmann S. M. Cowan D. A. (2022). Microbial biogeochemical cycling of nitrogen in arid ecosystems. Microbiol. Mol. Biol. Rev. 86:e0010921. doi: 10.1128/mmbr.00109-21, PMID: 35389249 PMC9199420

[ref55] Rozpądek P. Domka A. M. Nosek M. Ważny R. Jędrzejczyk R. J. Wiciarz M.. (2018). The role of strigolactone in the cross-talk between *Arabidopsis thaliana* and the endophytic fungus *Mucor* sp. Front. Microbiol. 9:441. doi: 10.3389/fmicb.2018.00441, PMID: 29615990 PMC5867299

[ref57] Song Y. Chen P. Xuan A. Bu C. Liu P. Ingvarsson P. K.. (2021). Integration of genome wide association studies and co-expression networks reveal roles of *PtoWRKY 42-PtoUGT76C1-1* in *trans*-zeatin metabolism and cytokinin sensitivity in poplar. New Phytol. 231, 1462–1477. doi: 10.1111/nph.17469, PMID: 33999454

[ref540] Song Y. Ci D. Tian M. Zhang D. (2016). Stable methylation of a non-coding RNA gene regulates gene expression in response to abiotic stress in Populus simonii. J. Exp. Bot. 67, 1477–1492. doi: 10.1093/jxb/erv543, PMID: 26712827

[ref58] Song Y. Li X. Yao S. Yang X. Jiang X. (2020). Correlations between soil metabolomics and bacterial community structures in the pepper rhizosphere under plastic greenhouse cultivation. Sci. Total Environ. 728:138439. doi: 10.1016/j.scitotenv.2020.138439, PMID: 32361108

[ref59] Su X. Su X. Yang S. Zhou G. Ni M. Wang C.. (2020). Drought changed soil organic carbon composition and bacterial carbon metabolizing patterns in a subtropical evergreen forest. Sci. Total Environ. 736:139568. doi: 10.1016/j.scitotenv.2020.139568, PMID: 32485376

[ref60] Sui X. Zhang R. Frey B. Yang L. Liu Y. Ni H.. (2021). Soil physicochemical properties drive the variation in soil microbial communities along a forest successional series in a degraded wetland in northeastern China. Ecol. Evol. 11, 2194–2208. doi: 10.1002/ece3.7184, PMID: 33717448 PMC7920768

[ref650] Sun N. Bu Y. Pan C. Wu X. Cao Y. Jing Y. (2021). Analyses of microstructure and dynamic deposition of cell wall components in xylem provide insights into differences between two black poplar cultivars. Forests 12:972. doi: 10.3390/f12080972, PMID: 33717448

[ref61] Tong S. Yuan M. Liu Y. Li X. Jin D. Cheng X.. (2021). Ergosterol-targeting fusion antifungal peptide significantly increases the Verticillium wilt resistance of cotton. Plant Biotechnol. J. 19, 926–936. doi: 10.1111/pbi.13517, PMID: 33217142 PMC8131044

[ref62] Tya B. Jxab C. Zzb C. Ywa B. (2021). Biochar-based fertilizer amendments improve the soil microbial community structure in a karst mountainous area. Sci. Total Environ. 794:148757. doi: 10.1016/j.scitotenv.2021.148757, PMID: 34225142

[ref63] Veach A. M. Chen H. Yang Z. K. Labbe A. D. Engle N. L. Tschaplinski T. J.. (2020). Plant hosts modify belowground microbial community response to extreme drought. mSystems 5:5. doi: 10.1128/mSystems.00092-20, PMID: 32606021 PMC7329318

[ref64] Veach A. M. Morris R. Yip D. Z. Yang Z. K. Engle N. L. Cregger M. A.. (2019). Rhizosphere microbiomes diverge among *Populus trichocarpa* plant-host genotypes and chemotypes, but it depends on soil origin. Microbiome 7:76. doi: 10.1186/s40168-019-0668-8, PMID: 31103040 PMC6525979

[ref65] Veyisoglu A. Tatar D. Saygin H. Inan K. Cetin D. Guven K.. (2016). *Microvirga makkahensis* sp. nov., and *microvirga arabica* sp. nov., isolated from sandy arid soil. Antonie Van Leeuwenhoek 109, 287–296. doi: 10.1007/s10482-015-0631-z, PMID: 26671415

[ref66] Vives-Peris V. de Ollas C. Gómez-Cadenas A. Pérez-Clemente R. M. (2020). Root exudates: from plant to rhizosphere and beyond. Plant Cell Rep. 39, 3–17. doi: 10.1007/s00299-019-02447-5, PMID: 31346716

[ref67] Wagg C. Bender S. F. Widmer F. van der Heijden M. G. A. (2014). Soil biodiversity and soil community composition determine ecosystem multifunctionality. Proc. Natl. Acad. Sci. 111, 5266–5270. doi: 10.1073/pnas.1320054111, PMID: 24639507 PMC3986181

[ref68] Wakabayashi T. Ishiwa S. Shida K. Motonami N. Suzuki H. Takikawa H.. (2021). Identification and characterization of sorgomol synthase in sorghum strigolactone biosynthesis. Plant Physiol. 185, 902–913. doi: 10.1093/plphys/kiaa113, PMID: 33793911 PMC8133691

[ref69] Wang Y. Hou L. Wu X. Zhu M. Dai Y. Zhao Y. (2021). Mycorrhiza helper bacterium *Bacillus pumilus* HR10 the improves growth and nutritional status of *Pinus thunbergii* by promoting mycorrhizal proliferation. Tree Physiol. 42, 907–918. doi: 10.1093/treephys/tpab139, PMID: 34730183

[ref70] Wang J. Liu L. Gao X. Hao J. Wang M. (2021). Elucidating the effect of biofertilizers on bacterial diversity in maize rhizosphere soil. PLoS One 16:e0249834. doi: 10.1371/journal.pone.0249834, PMID: 33891590 PMC8064744

[ref71] Wang W. Talide L. Viljamaa S. Niittylä T. (2022). Aspen growth is not limited by starch reserves. Curr. Biol. 32, 3619–3627.e4. doi: 10.1016/j.cub.2022.06.056 35820419

[ref72] Wanichthanarak K. Boonchai C. Kojonna T. Chadchawan S. Sangwongchai W. Thitisaksakul M. (2020). Deciphering rice metabolic flux reprograming under salinity stress via in silico metabolic modeling. Comput. Struct. Biotechnol. J. 18, 3555–3566. doi: 10.1016/j.csbj.2020.11.023, PMID: 33304454 PMC7708941

[ref73] Wu N. Li Z. Meng S. Wu F. (2021). Soil properties and microbial community in the rhizosphere of *Populus alba* var. *pyramidalis* along a chronosequence. Microbiol. Res. 250:126812. doi: 10.1016/j.micres.2021.126812, PMID: 34246038

[ref75] Wu X. Liu J. Meng Q. Fang S. Kang J. Guo Q. (2021). Differences in carbon and nitrogen metabolism between male and female *Populus cathayana* in response to deficient nitrogen. Tree Physiol. 41, 119–133. doi: 10.1093/treephys/tpaa108, PMID: 32822497

[ref76] Xing M. Wang Q. Li X. Li Y. Zhou X. (2021). Selection of keystone species based on stable carbon and nitrogen isotopes to construct a typical food web on the shore of Xingkai Lake, China. Ecol. Indic. 132:108263. doi: 10.1016/j.ecolind.2021.108263

[ref77] Ye X. Li Z. Luo X. Wang W. Li Y. Li R.. (2020). A predatory myxobacterium controls cucumber *fusarium* wilt by regulating the soil microbial community. Microbiome 8:49. doi: 10.1186/s40168-020-00824-x, PMID: 32252828 PMC7137222

[ref78] Yoneyama K. (2020). Recent progress in the chemistry and biochemistry of strigolactones. J. Pestic. Sci. 45, 45–53. doi: 10.1584/jpestics.D19-084, PMID: 32508512 PMC7251197

[ref81] Zhang Y. Zhao L. Zhao J. Li Y. Wang J. Guo R.. (2017). *S5H/DMR6* encodes a salicylic acid 5-hydroxylase that fine-tunes salicylic acid homeostasis. Plant Physiol. 175, 1082–1093. doi: 10.1104/pp.17.00695, PMID: 28899963 PMC5664462

[ref82] Zhang L. Zhou J. George T. S. Limpens E. Feng G. (2021). Arbuscular mycorrhizal fungi conducting the hyphosphere bacterial orchestra. Trends Plant Sci. 27, 402–411. doi: 10.1016/j.tplants.2021.10.008, PMID: 34782247

[ref83] Zhao M. Zhao J. Yuan J. Hale L. Wen T. Huang Q.. (2021). Root exudates drive soil-microbe-nutrient feedbacks in response to plant growth. Plant Cell Environ. 44, 613–628. doi: 10.1111/pce.13928, PMID: 33103781

[ref84] Zheng W. Zheng Q. Xue Y. Hu J. Gao M.-T. (2017). Influence of rice straw polyphenols on cellulase production by *Trichoderma reesei*. J. Biosci. Bioeng. 123, 731–738. doi: 10.1016/j.jbiosc.2017.01.009, PMID: 28202307

[ref85] Zhou S. Geng B. Li M. Li Z. Liu X. Guo H. (2021). Comprehensive analysis of environmental factors mediated microbial community succession in nitrogen conversion and utilization of ex situ fermentation system. Sci. Total Environ. 769:145219. doi: 10.1016/j.scitotenv.2021.145219, PMID: 33486184

[ref86] Zhou X. Wang J.-T. Zhang Z.-F. Li W. Chen W. Cai L. (2020). Microbiota in the rhizosphere and seed of rice from China, with reference to their transmission and biogeography. Front. Microbiol. 11:995. doi: 10.3389/fmicb.2020.00995, PMID: 32754120 PMC7365946

[ref87] Zhou X. Zhang X. Ma C. Wu F. Jin X. Dini-Andreote F.. (2022). Biochar amendment reduces cadmium uptake by stimulating cadmium-resistant PGPR in tomato rhizosphere. Chemosphere 307:136138. doi: 10.1016/j.chemosphere.2022.136138, PMID: 36002065

[ref88] Zhu Q. Yan K. Dong Y. Wang Y. (2022). Rhizosphere bacterial communities and soil nutrient conditions reveal sexual dimorphism of *Populus deltoides*. J. For. Res. 33, 1–11. doi: 10.1007/s11676-022-01517-x

