# Supplementary material for: Populus root exudates are associated with rhizosphere microbial communities and symbiotic patterns
Source: Front Microbiol. 2022 Dec 22;13:1042944. doi: 10.3389/fmicb.2022.1042944 (PMC9812961; doi:10.3389/fmicb.2022.1042944)
Supplement: Supplementary file 1 [file Data_Sheet_1.docx]

**Table. S1 The gradient elution conditions**

| Time/min | Mobile phase A% (water ＋ 0.1% formic acid) | Mobile phase B% (acetonitrile ＋ 0.1% formic acid) | Column temperature | Flow rate |
| --- | --- | --- | --- | --- |
| 0.01 | 95 | 5 | 40°C | 0.25 mL/min |
| 1.5 | 80 | 20 |  |  |
| 15 | 10 | 90 |  |  |
| 18 | 0 | 100 |  |  |
| 18.1 | 95 | 5 |  |  |
| 22 | 95 | 5 |  |  |


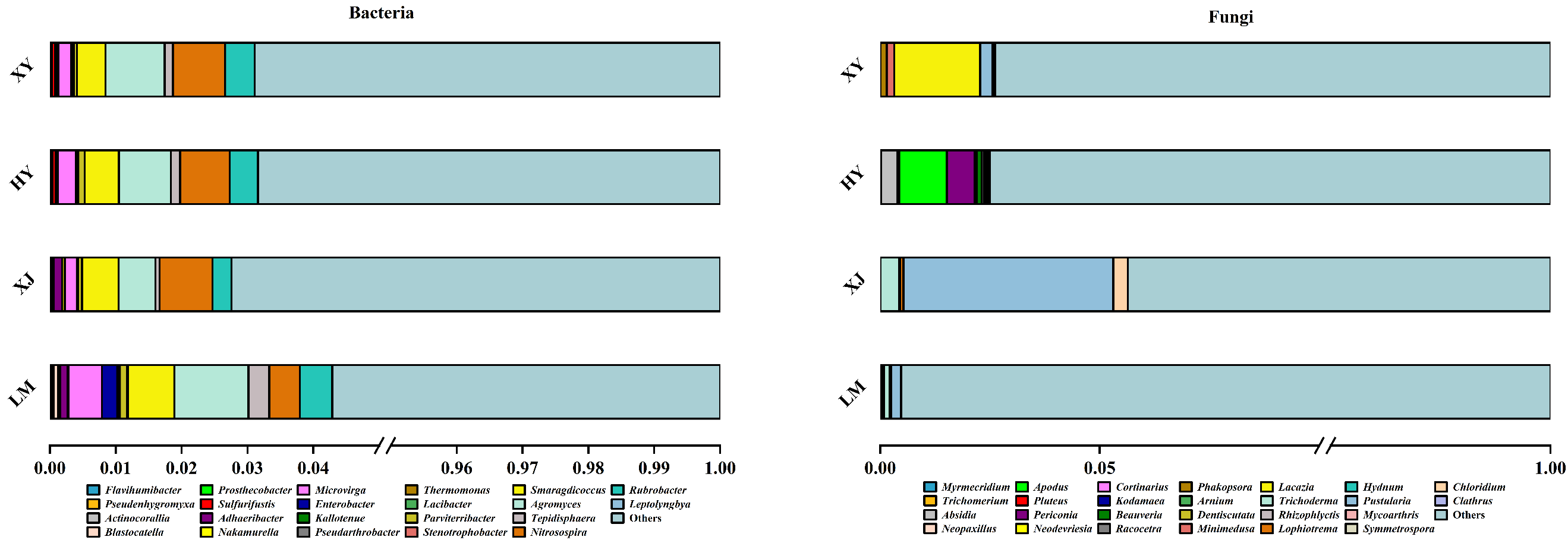


**Fig.** **S1** Rhizosphere keystone and others microbial community compositions of four *Populus* species at the genus level.

**Table. S2 The peak area of differential metabolites of four *Populus* species**

| Differential  metabolites | Mean value in each type of *Populus* | | | | Pair-wise test (*P*-value) | | | | | |
| --- | --- | --- | --- | --- | --- | --- | --- | --- | --- | --- |
|  | LM | XJ | HY | XY | LM vs  XJ | LM vs HY | LM vs XY | XJ  vs HY | XJ  vs XY | HY vs XY |
| Chrysin | 0.000±0.000 | 1.250±0.170 | 29.348±20.686 | 362.294±176.755 | 0.041 | 0.028 | 0.001 | 0.045 | 0.000 | 0.001 |
| Luvangetin | 8463.262±791.139 | 4171.554±161.516 | 11513.344±750.233 | 23666.135±1231.016 | 0.000 | 0.001 | 0.000 | 0.000 | 0.000 | 0.000 |
| Oleuropein | 17.356±5.709 | 435.355±22.632 | 79.344±5.573 | 104.369±9.892 | 0.000 | 0.000 | 0.000 | 0.000 | 0.000 | 0.011 |
| Salicylic acid | 213.105±11.562 | 275.985±40.937 | 458.952±38.372 | 336.125±1.119 | 0.006 | 0.000 | 0.000 | 0.000 | 0.013 | 0.004 |
| Strigol | 261.810±26.201 | 1.558±0.404 | 7.565±0.481 | 3.890±0.782 | 0.000 | 0.000 | 0.000 | 0.000 | 0.010 | 0.000 |
| Gentisic acid | 59.848±2.256 | 23.370±3.041 | 155.988±24.740 | 277.597±77.996 | 0.000 | 0.001 | 0.000 | 0.000 | 0.000 | 0.020 |
| Linoleic acid | 77.894±10.874 | 29.017±0.545 | 21.263±2.651 | 14.891±2.451 | 0.000 | 0.000 | 0.000 | 0.004 | 0.000 | 0.019 |
| Gemcitabine elaidate | 11.195±3.45 | 147.457±15.611 | 14.933±0.604 | 5.439±1.298 | 0.001 | 0.012 | 0.005 | 0.001 | 0.001 | 0.000 |
| Silandrone | 118.271±2.469 | 209.254±15.739 | 85.223±2.942 | 52.791±5.595 | 0.000 | 0.000 | 0.000 | 0.000 | 0.000 | 0.000 |
| Albendazole sulfoxide | 960.256±34.815 | 20590.235±1366.691 | 4218.527±190.901 | 6688.222±931.675 | 0.000 | 0.000 | 0.000 | 0.000 | 0.000 | 0.000 |
| Pentoxifylline | 305.952±54.764 | 415.608±63.114 | 931.315±80.586 | 160.651±27.686 | 0.035 | 0.001 | 0.004 | 0.003 | 0.000 | 0.000 |
| Clenbuterol | 82.323±9.704 | 262.494±14.027 | 187.099±15.657 | 103.321±2.998 | 0.000 | 0.000 | 0.007 | 0.000 | 0.000 | 0.000 |
| Triamifos | 59.61±2.183 | 247.655±29.888 | 202.831±7.541 | 103.284±8.086 | 0.000 | 0.000 | 0.000 | 0.010 | 0.000 | 0.000 |
| Aplindore | 25.798±4.990 | 178.274±21.952 | 50.802±4.569 | 82.346±14.361 | 0.000 | 0.002 | 0.000 | 0.000 | 0.000 | 0.031 |
| Idrocilamide | 213.794±12.780 | 272.722±24.591 | 126.662±20.968 | 172.793±2.290 | 0.002 | 0.000 | 0.013 | 0.000 | 0.000 | 0.025 |
| Alfatradiol | 132.531±14.767 | 58.894±6.631 | 47.369±1.329 | 84.112±9.584 | 0.009 | 0.005 | 0.037 | 0.007 | 0.002 | 0.000 |
| Nylidrin | 204.12±18.082 | 316.935±6.691 | 410.771±24.275 | 507.7±3.011 | 0.000 | 0.000 | 0.000 | 0.000 | 0.000 | 0.005 |
| Xibornol | 455.415±4.173 | 611.53±25.514 | 764.269±22.875 | 1137.787±20.152 | 0.000 | 0.000 | 0.000 | 0.010 | 0.000 | 0.000 |
| Gamabufagin | 83.647±9.456 | 53.069±4.185 | 237.395±1.091 | 127.644±6.525 | 0.002 | 0.000 | 0.002 | 0.000 | 0.000 | 0.000 |
| Ibufenac | 145.528±7.840 | 90.927±2.206 | 327.832±22.535 | 224.976±26.407 | 0.000 | 0.000 | 0.001 | 0.000 | 0.000 | 0.001 |
| Iloprost | 478.456±26.982 | 265.138±28.729 | 934.999±57.414 | 690.732±53.866 | 0.001 | 0.000 | 0.001 | 0.000 | 0.000 | 0.001 |
| N-Capryloyl-DL-homoserinelactone | 238.238±15.633 | 183.667±39.256 | 308.777±20.536 | 498.326±43.473 | 0.033 | 0.003 | 0.000 | 0.001 | 0.000 | 0.002 |
| Metacetamol | 14.943±1.836 | 27.838±0.787 | 8.324±0.355 | 82.261±12.687 | 0.000 | 0.004 | 0.000 | 0.000 | 0.000 | 0.000 |
| Fenclonine | 98.27±3.594 | 67.817±1.818 | 187.276±1.730 | 274.138±3.688 | 0.000 | 0.000 | 0.000 | 0.000 | 0.000 | 0.000 |
| Salmefamol | 271.001±13.298 | 5.565±1.421 | 48.936±4.237 | 25.037±11.570 | 0.000 | 0.000 | 0.000 | 0.000 | 0.012 | 0.004 |
| Calcipotriol | 1065.769±34.329 | 513.02±13.111 | 1718.207±116.643 | 2477.455±229.893 | 0.000 | 0.000 | 0.000 | 0.000 | 0.000 | 0.001 |
| Actarit | 160.588±35.853 | 293.848±21.917 | 369.265±44.798 | 1576.27±122.544 | 0.001 | 0.000 | 0.000 | 0.004 | 0.000 | 0.000 |
| Fenamiphos | 175.532±12.714 | 38.899±8.976 | 83.234±8.784 | 240.746±25.876 | 0.000 | 0.001 | 0.041 | 0.000 | 0.000 | 0.000 |
| Etridiazole | 47.239±1.229 | 86.403±14.069 | 71.456±11.718 | 599.009±60.094 | 0.001 | 0.025 | 0.000 | 0.038 | 0.000 | 0.000 |
| (1R, 5R, 11R, 12S, 14S, 17R, 20S, 21S)-21-Hydroxy-5,15-dimethyl-7-oxa-10-azaheptacyclo [12.6.2.0~1,11~.0~5,20~.0~6,10~.0~12,17~.0~17,21~] docos-15-en-19-one | 1628.957±129.911 | 536.598±22.289 | 2370.542±352.88 | 4027.377±316.079 | 0.005 | 0.030 | 0.000 | 0.000 | 0.000 | 0.001 |
| Binapacryl | 158.314±5.144 | 45.657±0.720 | 265.289±14.618 | 517.758±21.649 | 0.000 | 0.007 | 0.000 | 0.000 | 0.000 | 0.000 |
| Clindamycin  hydrochloride | 8.166±2.719 | 1.808±0.830 | 123.888±8.903 | 24.918±3.563 | 0.046 | 0.000 | 0.000 | 0.000 | 0.000 | 0.000 |
| Lidorestat | 40.83±14.852 | 12.854±5.38 | 111.013±18.232 | 179.095±41.845 | 0.008 | 0.000 | 0.000 | 0.000 | 0.000 | 0.005 |
| Methylenebis (phosphonic acid) - (~99~Tc) technetium (1:1) | 0.000±0.000 | 56.46±4.254 | 227.745±25.174 | 847.517±125.097 | 0.000 | 0.000 | 0.000 | 0.000 | 0.000 | 0.000 |
| Daminozide | 328.804±29.895 | 2.941±0.717 | 141.11±35.172 | 55.114±9.855 | 0.000 | 0.001 | 0.000 | 0.002 | 0.001 | 0.033 |
| Deltamethrin | 257.971±14.198 | 2.322±0.129 | 18.55±2.652 | 80.906±8.990 | 0.000 | 0.000 | 0.000 | 0.000 | 0.001 | 0.002 |
| N~2~, N~6~-Bis (2,3-dihydroxybenzoyl) -L-lysine | 0.000±0.000 | 31.21±14.948 | 175.983±20.823 | 1421.221±681.193 | 0.008 | 0.000 | 0.001 | 0.001 | 0.001 | 0.001 |
| Fluvalinate | 84.922±25.747 | 2.208±0.216 | 379.934±19.271 | 886.894±12.732 | 0.001 | 0.000 | 0.000 | 0.000 | 0.000 | 0.000 |
| Carbutamide | 14.535±3.192 | 835.404±71.842 | 52.201±5.181 | 27.168±3.398 | 0.000 | 0.000 | 0.013 | 0.000 | 0.000 | 0.003 |
